# Handheld Ultrasound Parameters of Lower Limb Muscles versus Bioelectrical Impedance Analysis Parameters for Skeletal Muscle Assessments in Arabic Female Adults

**DOI:** 10.3390/diagnostics14151582

**Published:** 2024-07-23

**Authors:** Nada H. Alamoudi, Dara Aldisi, Mohamed S. El-Sharkawy, Mahmoud M. A. Abulmeaty

**Affiliations:** 1 Community Health Sciences Department, College of Applied Medical Sciences, King Saud University, Riyadh 11362, Saudi Arabia; 444204060@student.ksu.edu.sa (N.H.A.); daldisi@ksu.edu.sa (D.A.); 2 Radiology and Medical Imaging Department, King Saud University, Riyadh 11922, Saudi Arabia; msherif@ksu.edu.sa

**Keywords:** ultrasounds, muscle mass, ASMM, FFM, medial gastrocnemius, rectus femoris

## Abstract

Ultrasound (US) is a promising tool for skeletal muscle assessment; however, US studies have scarcely focused on Arabic populations. This study examined the association of handheld US indicators and bioelectrical impedance analysis (BIA) parameters in healthy Arabic females. A cross-sectional study was conducted on 60 healthy Arabic females whose muscle thickness (MT) and cross-sectional area (CSA) of the rectus femoris (RF) were measured alongside their MT and pennation angle (PA) of the medial gastrocnemius (MG) muscle (both muscles on the dominant side). Anthropometric and body composition analyses quantified fat-free mass (FFM) and appendicular skeletal muscle mass (ASMM). Muscle strength was assessed using a handgrip dynamometer, and physical activity levels were recorded with the Global Physical Activity Questionnaire (GPAQ). The CSA of the RF and the MT of both the RF and MG correlated significantly with FFM and ASMM. The PA of MG showed no significant correlations with ASMM, FFM, or handgrip strength. The CSA of RF was significantly correlated with handgrip strength (*r* = 0.313, *p* = 0.015), while the PA of MG correlated positively with GPAQ score (*r* = 0.346, *p* = 0.007). The CSA of RF significantly predicted both ASMM (β = 0.883, *p* = 0.0002) and FFM (β = 1.935, *p* = 0.0001). In conclusion, handheld US parameters, especially the RF’s CSA, correlate with and can predict BIA-based FFM and ASMM in healthy females.

## 1. Introduction

Assessing muscle mass, strength, and function is vital for diagnosing and managing various medical conditions [1]. Based on current consensus definitions, the criterion of low muscle mass is considered a defining characteristic of malnutrition and sarcopenia [2]. Interestingly, research suggests that young women may be more susceptible to sarcopenia than young men [3].

Several established methods for measuring skeletal muscle mass, including computed tomography (CT) and magnetic resonance imaging (MRI), are considered gold standards due to their accuracy; however, they are limited by factors such as radiation exposure and high cost, thereby restricting their frequent use [4,5]. In addition, neither CT nor MRI has cutoff values at this time [6]. Even though dual-energy X-ray absorption (DXA) and bioelectrical impedance analysis (BIA) have been recommended as alternative skeletal muscle assessment methods by the European and Asian working groups for sarcopenia diagnosis (EWGSOP2 and AWGS), they are sometimes difficult to perform [7,8]. For example, patients with edema or any metal or device implants cannot undergo DXA or BIA [5]. In addition, these methods do not provide an index for muscle quality; however, they have cutoff values for muscle quantity [6].

Ultrasound (US) is a quick, affordable, and non-invasive muscle assessment method that is portable and accessible from the bedside [6,9]. In addition, US is an alternative measurement method that is valid and reliable for measuring local muscle mass [6].

Different muscle parameters can be estimated using US; for example, muscle quantity can be determined by some parameters, such as muscle thickness (MT) and cross-sectional area (CSA), while other parameters describe the muscle qualitatively, such as pennation angle (PA) [6]. US can assess both the quantity and quality of muscles, which represents an advantage over other imaging methods [10].

When it comes to muscle quantity, MT and CSA are significantly correlated with other primary approaches for examining muscle, such as MRI, CT, and DXA [10]. Despite US’s promising features for skeletal muscle assessment, US studies focused on healthy Arabic populations are lacking. The body composition and muscle strength of the Arabic population, especially those living in Saudi Arabia, are expected to differ from internationally published cutoffs. This study examined the association between handheld US indicators of lower limb muscles and BIA parameters in healthy Arabic females.

## 2. Materials and Methods

### 2.1. Study Design and Sample Selection

This cross-sectional study was conducted at the Applied Medical Sciences College clinics at King Saud University in Riyadh, Saudi Arabia, from December 2023 to March 2024. The participants included in the study were healthy adult Arabic females aged ≥18 years. Individuals with a body mass index (BMI) below 18.5 kg/m^2^ or exceeding 24.9 kg/m^2^, indicating underweight or overweight status, respectively, were excluded. Moreover, athletes who had at least two years of experience in their respective sports and engaged in consistent training sessions of 20 h or more per week were excluded [11]. Furthermore, pregnant women, participants taking weight-loss medication, or those with neuromuscular degenerative diseases, significant medical or metabolic conditions, or recent hospitalizations within the past 3–6 months were not included in the study. This study was approved by the Institutional Review Board (IRB) at King Saud University’s College of Medicine (Reference No. 23/0171/IRB-A) on 12 October 2023. Consent was obtained from all participants after they were fully informed about the study and its purpose. The sample size was estimated using the G*Power software (version 3.1.9.7) based on a point-biserial correlation test. We considered the correlations between several muscle measurements and the fat-free mass index (FFMI). The effect sizes were 0.371 for the MT of the rectus femoris muscle (RF), 0.404 for the CSA of the RF, and 0.390 for the MT of the medial gastrocnemius muscle (MG) [12]. The effect size for RF-MT (0.371) was the smallest; therefore, we input this value in G-Power to calculate the minimum sample size. Assuming a 5% significance level and 80% power, this resulted in an estimated minimum of 52 participants. We thought a response rate of 90% resulted in a total sample size of 58.

### 2.2. Anthropometric and Body Composition Measurements

Anthropometric measurements, including height, were manually measured using a Seca scale (Seca Co., Hamburg, Germany). Height results were approximated to the nearest centimeter (cm). Weight, appendicular skeletal muscle mass (ASMM), and fat-free mass (FFM) data were obtained using a segmental multifrequency bioelectrical impedance device (TANITA BC-418, Tanita Co., Tokyo, Japan). This device has eight electrodes and provides segmented information on five body parts: the arms, trunk, and legs. This device allowed us to calculate ASMM by summing the lean mass in the upper and lower limbs. To determine the appendicular skeletal muscle mass index (ASMMI), the ASMM (kg) was divided by the height squared (m^2^). To measure FFMI, FFM (kg) was divided by height squared (m^2^). BMI was calculated as weight divided by height squared (kg/m^2^).

### 2.3. Physical Activity Questionnaire

To subjectively estimate an individual’s level of physical activity, the Arabic version of the Global Physical Activity Questionnaire (GPAQ) (version 2.0) by the World Health Organization (WHO) was used [13]. The validity and reliability of the Arabic version of the questionnaire have been examined [14]. The GPAQ comprises 16 questions divided into three domains: work, transportation, and leisure time, aimed at capturing different aspects of physical activity, such as the frequency and duration of moderate- and vigorous-intensity activities [15]. Additionally, it includes an inquiry about sedentary behavior, specifically regarding daily sitting time [15]. The total GPAQ score is calculated by summing the total metabolic equivalent (MET)-minutes of activity for each domain. Individuals who achieve a minimum of 600 MET minutes per week are considered to meet the physical activity recommendations set by the WHO.

### 2.4. Muscle Strength Assessment

Muscle strength was assessed by measuring handgrip strength with a Jamar Hydraulic Dynamometer (Chicago, IL, USA). The participants were asked to squeeze the hydraulic dynamometer maximally. Three trials were performed for each participant’s dominant hand, and the mean value of the three measurements was used.

### 2.5. Ultrasound Measurements

Muscles were assessed ultrasonographically using the dual-head wireless handheld US scanner EagleView (Shenzhen, China) with a 10 MHz linear probe. All measurements were performed on the dominant side of the participants’ bodies and repeated three times. The mean values of these measurements were used. Participants were instructed to avoid physical activity for at least 30 min before the investigation.

All measurements were obtained using a generous amount of transmission gel. The probe was perpendicular to the skin, with the transducer exerting the least amount of pressure possible on the skin to minimize muscle compression. In addition, all measurement points were marked on the skin to ensure proper transducer placement when repeating the scan.

MT and CSA were measured in the RF muscle, while MT and PA were specifically measured in the MG muscle. The RF muscle was measured as the participant fully extended their limbs in a supine position at the midpoint between the greater trochanter and proximal patella border. The MG was measured as the participant was in a sitting position with their hips and knees at 90° angles, at a point approximately 30% of the way from the most medial point of the articular cleft of the knee to the most medial point of the top of the medial malleolus. These anatomical landmarks were identified following the protocol developed by the sarcopenia and ultrasound (SARCUS) working group [6].

**Figure 1:**
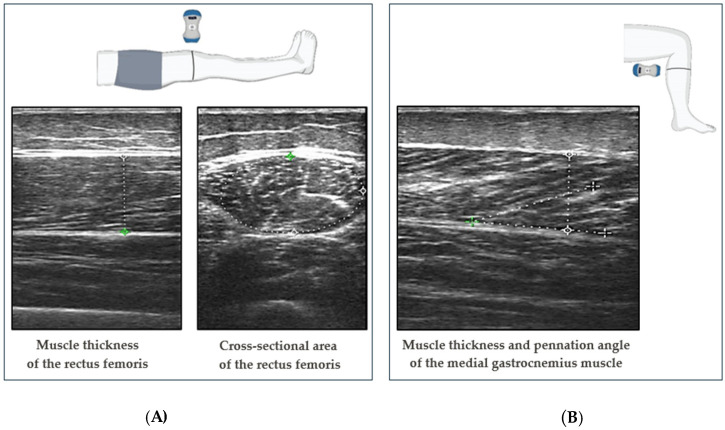
An illustration of the probe placement on the RF and MG and representative ultrasonography images of muscles. (**A**) MT is in the longitudinal section of the RF muscle, and CSA is in the transverse section of the RF muscle. (**B**) MT and PA in the longitudinal section of the MG muscle.

The images of the muscles on the US monitor were frozen when the investigator was satisfied with the probe’s position and the image’s quality. MT was defined as the distance between the superficial fascia and the deep fasciae of the muscle. CSA was measured by outlining the muscle border with a best-fit ellipse. MT was evaluated with the probe placed longitudinally, while CSA was measured with the probe placed transversely. PA was defined as the angle between the muscle fibers and the deep fascia of the muscle. Lastly, PA was measured with a transducer probe longitudinally aligned with the muscle fiber fascicles (Figure 1).

### 2.6. Statistical Analysis

The statistical analysis was conducted using the SPSS software (version 29). The normality of the data was assessed using the Shapiro–Wilk test. Mean ± standard deviation (SD) was used to express continuous variables with a normal distribution, while median and interquartile range (IQR) were used for non-normally distributed data. Categorical variables were presented as numbers (n) and percentages (%). Correlations between continuous variables were examined using Spearman’s correlation coefficient and Pearson correlation analysis as appropriate. The interpretation of the Spearman’s rank correlation coefficient and Pearson correlation was categorized as poor (≥0.1 and <0.3), fair (≥0.3 and <0.6), moderate (≥0.6 and <0.8), very strong (≥0.8 and <1), and perfect (=1) [16]. Given the high correlation (0.79) between RF-CSA and RF-MT, RF-MT was excluded to avoid multicollinearity. Multiple linear regression models were used to examine the relationship between the independent variables (the CSA of the RF muscle and the MT of the MG muscle) and the dependent variables (ASMM and FFM).

## 3. Results

### 3.1. Participant Characteristics

The study population included 60 Saudi adult females aged between 18 and 46 years (median, 21 years; IQR, 3 years). The studied females had an average weight of 53.78 ± 6.65 kg and an average height of 157.73 cm. The median BMI was 21.29 kg/m^2^, indicating relatively normal weight. The mean ASMM and FFM were 15.72 kg and 37.92 kg, respectively (Table 1).

### 3.2. Association between Ultrasound Parameters and Body Composition Analysis Parameters

**Figure 2:**
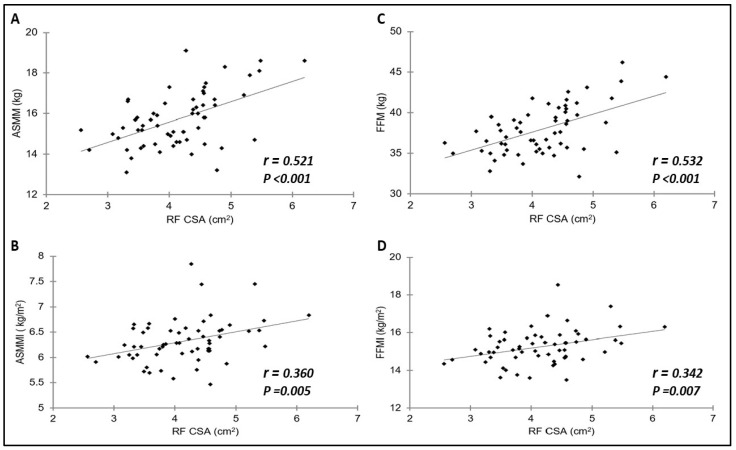
Correlations of RF-CSA with ASMM (**A**), ASMMI (**B**), FFM (**C**), and FFMI (**D**).

The correlation coefficients between ASMM, ASMMI, FFM, and FFMI and RF-CSA, RF-MT, MG-MT, and MG-PA are shown in Figure 2 and Table 2. Unlike PA, CSA and MT were significantly correlated with ASMM, ASMMI, FFM, or FFMI for both RF and MG muscles. In the RF, significant correlations were found between the CSA and ASMM (*r* = 0.521) and FFM (*r* = 0.532), all with *p* < 0.001. The MT exhibited positive significant correlations with all four muscle parameters (ASMM, ASMMI, FFM, FFMI) for both the RF and MG muscles.

### 3.3. Association between Ultrasound Parameters, Handgrip Strength, and Physical Activity Level

The correlation analysis examined the relationship between US parameters, handgrip strength, and physical activity level. Significant positive correlations were found between the RF-CSA and HGS (*r* = 0.313, *p* = 0.015). Moreover, positive correlations were observed between the MG-PA and GPAQ scores and between the RF-MT and GPAQ scores, as shown in Table 3.

### 3.4. Factors Influencing ASMM and FFM Determined via Multiple Linear Regression Analysis

Table 4 summarizes the results of multiple linear regression analyses conducted to identify the factors influencing ASMM. The results indicated that RF-CSA significantly predicted ASMM (β = 0.883 95% CI [0.443, 1.332], *p* = 0.0002), whereas MG-MT did not show a significant predictor of ASMM. The model accounted for 31.2% of the variance in ASMM (*p* < 0.001).

Additionally, a multiple linear regression analysis was conducted to predict FFM (Table 5). RF-CSA (β = 1.935, *p* = 0.0001) and MG-MT (β = 0.433, *p* = 0.039) were significant predictors of FFM. Overall, the model explained 33.4% of the variability in FFM.

## 4. Discussion

The main findings of this study include significant correlations between US parameters, specifically CSA and MT, for both the RF and MG muscles and BIA-derived muscle parameters. The MG’s PA had a significant positive correlation with the GPAQ scores. Furthermore, the RF-CSA consistently exhibited significant predictive power for both ASMM and FFM.

Several studies have shown a significant association between quantitative US parameters and BIA-derived muscle parameters, which is consistent with our results [12,17,18,19,20,21]. In this study, RF-MT and RF-CSA showed significant correlations with FFMI. These results align with the findings of a previous study that investigated 313 geriatric outpatients and reported significant correlations between RF-MT and FFMI (*r* = 0.371, *p* < 0.001) and RF-CSA and FFMI (*r* = 0.404, *p* < 0.001) [12]. Moreover, the identification of a positive correlation between ASMM and RF-MT is consistent with a prior study conducted among 121 females (*r* = 0.234, *p* = 0.010) [18].

The correlation between RF-MT and ASMMI identified in the current study was weaker than that reported in previous studies. For example, one study observed a significantly stronger correlation in heart failure patients’ right-side RF, while another study found a stronger correlation among 40 stroke patients’ nonparetic-side RF [19,21]. It is worth noting that both these studies measured RF-MT transversely to the muscle [19,21]. The disparity in correlation strength could stem from variations in the sample population (patients vs. healthy individuals) or MT probe positioning (longitudinal vs. transverse). For example, one study found that FFMI significantly correlated with CSA-RF in chronic obstructive pulmonary disease patients; however, this link was absent in healthy controls [22]. Similarly, recent research has investigated how the probe position affects the link between vastus lateralis MT and lower limb muscle mass, finding a stronger association with transverse probe placement compared to a more moderate association with longitudinal placement [23].This suggests that transverse positioning may be preferable for MT measurement when advanced tools are unavailable.

Moreover, we observed a significant correlation between MG-MT and ASMMI, which aligns with the findings from another study [21]. Additionally, a study involving 193 older Japanese adults reported a significant association between MG-MT, measured vertically with the probe positioned at the maximal point of the below-knee circumference on the right side, and ASMMI (*r* = 0.51, *p* < 0.001) [20].

Furthermore, the significant correlation identified between MG-MT and FFMI aligns with the findings of another study that also identified a positive and significant correlation between MG-MT and FFMI [12]. Notably, the measurement protocol employed in Ozturk et al.’s study closely aligns with our work for MG-MT measurement, strengthening our findings and suggesting reproducibility and validity in our methodology.

Additionally, a study involving 100 older adult subjects found significant correlations between MG-MT and FFMI [17]. These measurements were obtained while the participants lay prone with their legs extended to capture images of the point of maximal bulkiness of the MG muscle [17]. These findings collectively support the notion of a robust association between MG-MT and FFMI across different populations and measurement protocols. The consistency in the results across studies, despite variations in sample characteristics and measurement techniques, underscores the reliability of the observed correlation.

Our study found no significant correlations between MG-PA and the muscle parameters derived from BIA. This finding aligns with another study that observed no significant association between MG-PA and appendicular lean mass derived from DXA [24]. However, another study identified a weak negative correlation (*r* = −0.231, *p* = 0.035) between left-leg MG-PA and FFMI, but no correlation for right-leg MG-PA [17].

We found a significant positive correlation between MG-PA and overall physical activity levels, suggesting a potential link between muscle architecture and physical activity engagement. These results align with studies demonstrating associations between MG-PA and functional outcomes such as swimming performance and gait speed [24,25]. Our findings contribute to this body of evidence by highlighting the potential utility of MG-PA as a marker of physical activity levels.

Although MG-PA is often used to assess muscle architecture, which is thought to be linked to strength generation [10], our findings align with previous studies that identified no significant correlation between MG-PA and HGS. One investigation involving 100 elderly participants revealed no significant correlation between MG-PA and HGS [17]. Another study conducted among 265 elderly Chinese individuals found that MG-PA on the right side did not significantly correlate with dominant HGS [24]. This suggests that MG-PA may not directly reflect upper body strength. Therefore, these results indicate that although MG-PA may not directly impact muscle parameters such as FFM, ASMM, and upper body strength, it may still be associated with physical activity levels.

The significant correlation between HGS and RF-CSA on the dominant side aligns with previous research [12], highlighting the potential role of RF-CSA as a predictor of upper body strength. This finding contributes to the growing body of evidence supporting the use of US-derived muscle parameters in assessing musculoskeletal health.

The multiple linear regression analysis revealed RF-CSA to be a significant predictor of ASMM, which aligns with the findings of Wilkinson et al., who found RF-CSA to be a moderate predictor of ASMM among chronic kidney disease patients [26]. However, the exclusive predictive power of MG-MT for FFM suggests potential differential roles of these muscle components in body composition. Notably, a study among healthy Japanese individuals aged ≥ 65 years identified MG as a significant predictor for low ASMMI (β = −0.539, *p* < 0.01) [5]. Furthermore, another study suggested that variations in gender, BMI, and MG-MT could explain 80% of the variation in ASMMI [20].

Although our analysis focused on specific muscle sites to predict ASMM and FFM, the RF muscle warrants particular attention due to its superficial accessibility for US imaging and its susceptibility to age-related muscle loss, which typically affects the front of the thigh muscles first [27]. Nonetheless, the potential of other muscle sites for assessing muscle mass cannot be overlooked, as evidenced by studies incorporating multiple sites for estimating lean body mass. For instance, Abe et al. proposed a time-intensive approach utilizing whole-body multisite US to estimate lean body mass, incorporating MT values from nine specific sites (forearm, biceps, triceps, abdomen, subscapular, quadriceps, hamstrings, gastrocnemius, and tibialis anterior muscles) as parameters in the equation [28]. Moreover, another study suggested using US to measure MT at four sites (anterior upper arm, anterior and posterior thigh, and posterior lower leg) along with limb length to predict DXA-based FFM [29].

Similarly, specific muscle assessment has been emphasized in other studies. One such study suggested that ASMM estimation can be effectively achieved by evaluating only two muscles (RF and biceps brachii), particularly in females [18]. However, evidence has also suggested that a simple US of the forearm may sufficiently predict DXA-based appendicular lean mass [30].

These findings highlight the importance of integrating US-derived metrics as adjunctive tools for estimating ASMM and FFM. This holds considerable promise for both clinical practice and research endeavors. In addition, this study underscores the positive links between US quantitative parameters and BIA-derived muscle parameters, emphasizing the need for standardized protocols in US measurements of MT and CSA. Although using the US for nutritional assessment is promising, establishing cutoff values, including ethnic and sex-specific values, is essential. Educating dietitians on US-based muscle assessment and fostering collaborative research would accelerate progress in this field and clinical applications.

## 5. Conclusions

Our findings in healthy Arabic females suggest a promising role for the handheld US, especially RF-CSA, in predicting BIA-derived estimates of ASMM and FFM. Future research should explore whether these findings are generalizable to more diverse populations.

## Figures and Tables

**Table 1:** Participant demographics and clinical characteristics.

Variables	Total Sample (n = 60)
Age (years)	21 (3)
Weight (kg)	53.78 ± 6.65
Height (cm)	157.73 ± 6.08
BMI (kg/m^2^)	21.29 (3.196)
ASMM (kg)	15.72 ± 1.37
ASMMI (kg/m^2^)	6.26 (0.49)
FFM (kg)	37.92 ± 2.99
FFMI (kg/m^2^)	15.25 ± 0.91
RF-CSA (cm^2^)	4.14 ± 0.72
RF-MT (mm)	16.11 ± 2.21
MG-MT (mm)	15.55 ± 1.64
MG-PA (°)	32.45 ± 4.77
Dominant Average hand grip (kg)	19.19 ± 3.61
GPAQ score (MET-min/week)	1640.50 (3417)
Recommended GPAQ ≥ 600 MET-min/week	
Yes	40 (66.7%)
No	20 (33.3%)

Based on normality, data were presented as n (%), mean ± SD, or median (IQR). BMI: Body mass index; ASMM: Appendicular skeletal muscle mass; ASMMI: Appendicular skeletal muscle mass index; FFM: Fat-free mass; FFMI: Fat-free mass index; RF: Rectus femoris; CSA: Cross-sectional area; MT: Muscle thickness; MG: Medial gastrocnemius; PA: Pennation angle; GPAQ: Global Physical Activity Questionnaire.

**Table 2:** Correlations between ultrasound parameters and ASMM, ASMMI, FFM, and FFMI.

	ASMM (kg)		ASMMI (kg/m^2^)		FFM (kg)		FFMI (kg/m^2^)	
	*r*	*p*-Value	*r*	*p*-Value	*r*	*p*-Value	*r*	*p*-Value
RF-MT (mm)	0.393 **	0.002	0.340 *	0.008	0.417 **	<0.001	0.378 **	0.003
MG-MT (mm)	0.342 **	0.007	0.432 **	<0.001	0.370 **	0.004	0.440 **	<0.001
MG-PA (°)	0.070	0.595	0.133	0.322	0.085	0.518	0.200	0.126

ASMM: Appendicular skeletal muscle mass; ASMMI: Appendicular skeletal muscle mass index; FFM: Fat-free mass; FFMI: Fat-free mass index; RF: Rectus femoris; MT: Muscle thickness; MG: Medial gastrocnemius; PA: Pennation angle. ** Correlation is significant at the 0.01 level (2-tailed), * Correlation is significant at the 0.05 level (2-tailed).

**Table 3:** Correlation analysis of ultrasound parameters with handgrip and physical activity level.

	Average HGS (kg)		GPAQ Score (MET-min/Week)	
	*r*	*p*-Value	*r*	*p*-Value
RF-CSA (cm^2^)	0.313 *	0.015	0.126	0.338
RF-MT (mm)	0.185	0.157	0.275 *	0.033
MG-MT (mm)	0.091	0.487	0.172	0.190
MG-PA (°)	−0.019	0.883	0.346 **	0.007

HGS: Handgrip strength; GPAQ: Global physical activity questionnaire; RF: Rectus femoris; CSA: Cross-sectional area; MT: Muscle thickness; MG: Medial gastrocnemius; PA: Pennation angle. ** Correlation is significant at the 0.01 level (2-tailed), * Correlation is significant at the 0.05 level (2-tailed).

**Table 4:** Regression analysis of factors influencing ASMM.

Independent Variables	R^2^	Standardized β	95% CI		*p*-Value
			Lower Bound	Upper Bound	
RF-CSA	0.312	0.883	0.443	1.322	0.0002
MG-MT		0.176	−0.016	0.368	0.071

Dependent Variable is the ASMM; RF: Rectus femoris; CSA: Cross-sectional area; MT: Muscle thickness; MG: Medial gastrocnemius.

**Table 5:** Regression analysis of factors influencing FFM.

Independent Variables	R^2^	Standardized β	95% CI		*p*-Value
			Lower Bound	Upper Bound	
RF-CSA	0.334	1.935	0.993	2.878	0.0001
MG-MT		0.433	0.022	0.845	0.039

Dependent Variable is the FFM; RF: Rectus femoris; CSA: Cross-sectional area; MT: Muscle thickness; MG: Medial gastrocnemius.

## Data Availability

Data are available to the corresponding author upon reasonable request.
